# Prevalence of *Leptospira* spp. in Lithuanian Wild Boars (*Sus scrofa*)

**DOI:** 10.3390/pathogens14010085

**Published:** 2025-01-16

**Authors:** Birutė Karvelienė, Inga Stadalienė, Jūratė Rudejevienė, Evelina Burbaitė, Dalia Juodžentė, Marius Masiulis, Jūratė Buitkuvienė, Jurgita Šakalienė, Gintaras Zamokas

**Affiliations:** 1 Dr. L. Kriaučeliūnas Small Animal Clinic, Faculty of Veterinary, Veterinary Academy, Lithuanian University of Health Sciences, 47181 Kaunas, Lithuania; 2 Neurology and Neurosurgery Division, San Marco Veterinary Clinic, 35030 Veggiano, Italy; 3 National Food and Veterinary Risk Assessment Institute, 08409 Vilnius, Lithuania

**Keywords:** *Leptospira*, wild boar, Lithuania, serology

## Abstract

*Leptospira* is a bacteria responsible for a widespread zoonosis that affects both humans and animals. Leptospirosis is a challenging pathology to diagnose and treat since its signs are unspecific and symptoms vary greatly. The disease seems to be highly prevalent in environments where reservoir animals such as rats and small mammals are common. Even though leptospirosis in humans in Lithuania is rare, it remains a disease of significance in Europe. Information on reservoir animals and prevalence of *Leptospira* in wild animals in Lithuania is lacking. The aim of this country-wide study was to evaluate the seroprevalence of *Leptospira* in wild boars in Lithuania. Hunted animals were collected from ten counties that represented the boar population of the country. The sera of 451 collected boars were evaluated for eight *Leptospira* serovars using the microscopic agglutination test. Seropositivity was observed in 102 (22.6%) boars. Overall, 194 positive reactions occurred. Boars older than 2 years were affected by more serovars and were more seropositive than younger boars (*p* < 0.05). The highest number of positive reactions was observed in Panevėžys (87.9%) and Vilnius (69.1%) counties. The results of this study might indicate that the wild boar is a reservoir animal of *Leptospira* and plays a role in its transmission in Lithuania.

## 1. Introduction

*Leptospira* was first described in 1907 and has been studied ever since [1]. Spirochetes are the causatives of a zoonotic leptospirosis disease in humans and infection in animals that manifests with a wide variety of clinical presentations [1,2,3,4]. In humans, leptospirosis has been recognized as an important cause of febrile disease, renal failure, and jaundice [5,6,7,8]. In dogs, all organ systems can be affected, from acute kidney injury to ophthalmologic, dermatologic, and reproductive tract involvement [4,9]. In farm animals, abortions and infertility cases are as common as reduced production of milk, resulting in economic losses [6,10,11]. Reservoir animals, such as rats, mice, opossums, bats, or even cattle, are usually asymptomatic, but play an important role in the transmission of *Leptospira*, shedding the bacteria in their urine [2,6,12,13]. Multiple risk factors have been established for contracting leptospirosis. The most significant one is being in close contact with reservoir animals, especially rats [1,13,14]. Equally as important is occupational hazard, as *Leptospira* thrives in certain locations such as humid soils and water bodies. Being a hunter, butcher, farmer, veterinarian or working in agriculture increases the risk of being in close contact to the causative of the disease [2,7,15,16]. A recent seroepidemiological study of hunters in Austria has concluded that as many as 10% of hunters [17] and 75% of hound dogs in Japan [18] were seropositive for *Leptospira*. A higher risk of infection was also observed in socioeconomically challenged areas [15,16,19]. It is speculated that communities affected by poverty are more likely to be in contact with rural areas that contain small mammals, rodents, and other *Leptospira* reservoir animals.

The prevalence of leptospirosis in humans varies greatly based on geographical location. The United States Centers for Disease Control and Prevention has estimated that a million cases of human leptospirosis occur worldwide yearly [20]. Its prevalence in Europe is considerably lower. The data of the European Centre for Disease Prevention and Control (ECDC) state that 1261 cases of leptospirosis were reported in Europe in 2023, 14 of which were fatal [21]. This disease is not common in Lithuania and the last case was recorded in 2021 [22,23]. However, information on *Leptospira*, its prevalence, possible reservoir animals, and risk factors in Lithuania is lacking. The aim of this study was to evaluate the prevalence of *Leptospira* antibodies in the population of wild boars (*Sus scrofa*) in Lithuania. We hypothesized that even though human leptospirosis is rare, boars could be reservoir animals.

## 2. Materials and Methods

This study was approved by the Ethics Committee of the Lithuanian University of Health Sciences with the license number 2024-BEC3-T-034, issued on the 2nd of December, 2024.

### 2.1. Sample Collection

This study was conducted during the wild boar hunting season of the year 2021 in the months from May to November in 10 different counties that represent the country as a whole. All animals were hunted in adherence to the national hunting law, with the purpose of controlling the population. None of the animals were specifically hunted for the conduction of this study. The collected blood samples were also used for national African swine fever disease control and assessment. Responsible veterinary doctors from the State Food and Veterinary Service collected blood samples from the hunted wild boars using sterile vacuum tubes free of anticoagulant. The samples were transported in ice-cooled containers to the National Food and Veterinary Risk Assessment Institute‘s Serology Unit, accompanied by documentation indicating the location, county code, gender, and age of each hunted wild boar. The samples were centrifuged, and the obtained separated sera were stored at −20 °C until further analysis. For each hunted wild boar, its age group was determined by evaluating the degree of tooth eruption and wear of the lower jaw teeth, as reported previously [24]. Three age groups were identified: juvenile (up to 12 months old), sub-adult (12–24 months old), and adult (over 24 months old).

### 2.2. Laboratory Testing

Leptospirosis was detected using the microscopic agglutination test (MAT) with live leptospiral cultures to detect antibodies against specific serogroups. The standard methodology used for this study adhered to the guidelines as proposed by the OIE (World Organization for Animal Health) *Manual of Standards for Diagnostic Tests and Vaccines*, Chapter 3.1.12. Leptospirosis, Serological tests [25]. The antigens used for serogroup 8 detection were as follows: Leptospira Bratislava (strain Jez Bratislava); L. Canicola (strain Hond Utrecht IV); L. Saxcoebing (strain Mus 24); L. Copenhageni (strain M-20); L. Grippotyphosa (strain Andaman); L. Pomona (strain Pomona); L. Sejroe (strain M 84); and L. Tarassovi (strain Perepelicin). The MAT was conducted using microplates with duplicate dilutions of 1:50 and 1:100. However, only the results from the 1:100 dilution were considered to be significant and further analyzed. The reaction was assessed using a dark-field microscopy to evaluate the degree of agglutination. A four-plus scale was used. A positive reaction was defined as a 2+ agglutination (50%) in the 1:100 dilution.

### 2.3. Statistical Analysis

The data were recorded and analyzed with Microsoft Office Excel (for Microsoft 365 MSO, Version 2412). Additional statistical data analysis was performed using the IBM SPSS Statistics^®^ software package (Statistical Package for Social Sciences 29 for Windows, version 29). Boar groups are described using the number (*n*) of boars within a certain group, and seropositive percentage (%) (i.e., seropositivity by month is described by the number of seropositive boars/number of boars hunted that month). Relationships between boar age groups, sex, and county were evaluated using the Chi-square (χ^2^) test. A Confidence Interval (CI) of 95% was used in the statistical analysis. Differences were considered statistically significant when *p* < 0.05.

## 3. Results

In total, sera samples from 451 boars were collected and examined. Male boars were more common (342/451; 75.9%) than females (109/451; 24.2%) in our study. The toungest boars (up to 12 months of age) were the least common (30/451; 6.7%). About half of the boars (233/451; 51.7%) were 12–24-month-olds and the remainder (188/451; 41.7%) were older than 24 months. Boars were hunted in 10 counties: Vilnius (55; 12.2%), Kaunas (60; 13.3%), Klaipėda (42; 9.3%), Šiauliai (56; 12.4%), Panevėžys (33; 7.3%), Marijampolė (15; 3.3%), Telšiai (56; 12.4%), Tauragė (41; 9.1%), Alytus (37; 8.2%), and Utena (56; 12.4%). Hunting took place in the months from May to November. In May, 118 (26.2%) boars were hunted and enrolled in the study; in June, 191 (42.4%); July, 97 (21.5%), September, 11 (2.4%); October, 31 (6.9%); November, 3 (0.7%). None of the boar samples were examined in August, as it is a month with significant hunting restrictions in Lithuania. Almost two-thirds (68.5%) of the boars were hunted in May and June.

Within the study, boar seropositivity and the number of positive reactions were described as different entities. Overall seropositivity was observed in 102 (22.6%) boars, but 194 positive reactions were observed. Of the seropositive boars, 65 (63.7%) were positive for one serovar and 37 (36.3%) were positive for multiple pathogens. Of boars that were seropositive for multiple serovars, 12 (32.4%) tested positive for two serovars, 13 (35.1%) for three serovars, 5 (13.5%) for four serovars, 1 (2.7%) for five serovars, 3 (8.1%) for six serovars, 2 (5.4%) for seven serovars, and 1 (2.7%) for eight serovars. Seropositivity for serovars (sv.) was as follows: Copenhageni 44 (9.8%), Bratislava 42 (9.3%), Canicola 37 (8.2%), Sejroe 21 (4.7%), Tarassovi 15 (3.3%), Pomona 13 (2.9%), Grippotyphosa 12 (2.7%), Saxkoebing 10 (2.2%).

No statistical difference was observed between sexes: males were positive in 72/342 cases (21.1%) and females were positive in 30/109 cases (27.5%) (*p* > 0.05). Within age groups, seropositivity was lowest in boars up to 12 months old (5/30; 16.7%). Boars from 12 to 24 months old were slightly more seropositive (41/233; 17.6%). Boars older than 24 months were the most seropositive (56/188; 29.8%). This finding was statistically significant (*p* < 0.05). Seropositivity for the serovars Saxkoebing, Canicola, and Bratislava was significantly more common in older boars (*p* < 0.05). In terms of the number of positive reactions, the youngest boars had 10 positive reactions (5.2%), the middle age group had 73 positive reactions (37.6%), and boars older than 2 years had 111 positive reactions (57.2%). Out of all the positive reactions, 57.2% belonged to boars older than 24 months. Boars older than 2 years were statistically significantly affected by more serovars and more seropositive than younger boars (*p* < 0.05). The results are summarized in Table 1.

Seropositivity varied statistically significantly in different months and peaked in September (*p* < 0.05). Seropositivity by month was as follows: May, 14/118 (11.9%); June, 24/191 (12.6%); July, 42/97 (43.3%); September, 6/11 (54.5%); October, 16/31 (51.6%); November, 0/3 (0%).

**Figure 1:**
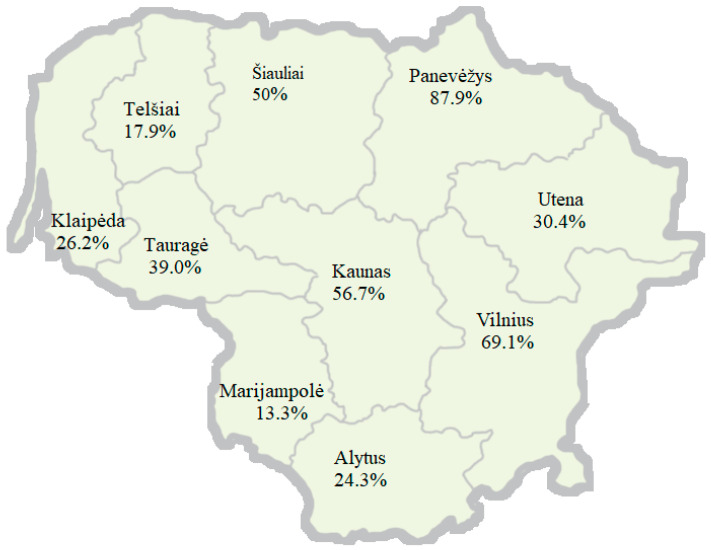
Percentage of seropositive reactions (%) in 10 Lithuanian counties.

Boar seropositivity within the counties was as follows: Vilnius, 18/55 (32.7%); Kaunas, 15/60 (25.0%); Klaipėda, 7/42 (16.7%); Šiauliai, 17/56 (30.4%); Panevėžys, 8/33 (24.2%); Marijampolė, 2/15 (13.3%); Telšiai, 9/56 (16.1%); Tauragė, 7/41 (17.1%); Alytu, 6/37 (16.2%); Utena, 13/56 (23.2%). No significant associations between distribution for serovars and county were observed. The number of positive reactions for different serovars in the counties is summarized in Figure 1 and Table 2. The highest numbers of positive reactions were observed in Panevėžys (87.9%) and Vilnius (69.1%) counties. The fewest numbers of positive reactions were found in Marijampolė (13.3%) and Telšiai (17.9%) counties.

## 4. Discussion

*Leptospira* is an organism responsible for a zoonosis dangerous to both animals and humans. Species such as mice, rats, boars, deer, and other mammals are thought to be reservoir animals that continue to spread the pathogen by shedding bacteria in their urine [5,26,27,28]. In Lithuania, the wild boar population is carefully monitored, as African swine fever is a disease of increasing importance [29]. It is estimated that 20–30 thousand boars are hunted yearly to limit their spread into rural areas, where they are capable of transmitting various pathogens and becoming a potential health hazard to both humans and other animals.

Multiple studies have already been performed worldwide to evaluate seroprevalence of *Leptospira* in boars and the data is variable [30,31,32,33,34,35,36,37,38,39,40,41,42,43]. The main findings of boar *Leptospira* seroprevalence in different countries are summarized in Table 3. In Portugal, the seroprevalence seems to be the highest (65.4%) [37]. In Slovenia, it reached 45.5% [41]; in Croatia it ranged from 26 to 31.9% [31,35]; in Brazil, 20.5% [33]; in France, 18.4% [38]; in Germany, 17.7% [34]; in the Czech Republic, 16.9% [36]; in Spain, 14.6% [43]; in Italy it varied 6–15.3% [30,32,42]; in Poland, 10.4% [39]; and in Sweden, 3.1% [40]. In our study, it was established to be 22.6%. In the context of the current data in the European continent, the *Leptospira* seroprevalence in the boars of Lithuania is considered high.

In this study, boar sera were tested for eight chosen *Leptospira* serovars. However, new serovars that can potentially affect people and animals are emerging. For instance, when *Leptospira wolffii* was first detected in a dog, it was determined that it was a predominant species affecting 93% of tested dogs in Iran [44]. To minimize the risk of dogs becoming a host animal and shedding bacteria in a household environment, preventative measures have been taken. In Europe and North America, vaccines have been used for almost half a century and dog seroprevalence is successfully decreasing [4,45]. However, vaccination does not prevent disease completely and protocols are not uniform around the globe. Vaccines can contain from one to four different serovars, depending on the geographic location [4,46]. It was found, however, that even immunized animals can be infected if prevention is not properly conducted [47]. Vaccinated dogs, therefore, may be susceptible to novel serovars not found in the vaccines. Constant efforts are being made to establish which serovars are the most relevant clinically and if collected evidence is sufficient to include certain *Leptospira* serovars in the vaccines [4,45,48]. Consequently, understanding the distribution of serovars within different countries is of incredible importance.

To date, in Lithuania, several epidemiological studies have been performed to detect *Leptospira* (in small rodents, swine, horses, and cattle) and to establish possible reservoir animals [49,50,51,52]. Only 4.4% of small rodents, such as mice and voles had Leptospiral DNA, and L. Kirschneri was isolated from them [49]. Swine were mostly seropositive for serovars Bratislava and Pomona [50]. Prevalence in horses was 18.6%, and they were mostly affected by serovars Canicola and Copenhageni [51]. Cattle were most seropositive for serovar Grippotyphosa and total seroprevalence for Leptospira was 7% [52]. Our study results indicate that boars in Lithuania are mostly affected by serovars Copenhageni and Bratislava. Based on the current literature and our findings, it is speculated that serovars Copenhageni, Bratislava, and Canicola are common serovars among reservoir animals in Lithuania [50,51].

The boar seroprevalence between sexes did not differ. However, in accordance with recent literature [35,36,37,42], the oldest boars (older than 24 months) were the most affected in this study. Boars older than 2 years were statistically significantly affected by more serovars and were more seropositive than younger boars. This could be explained by several factors. Older individuals have a longer cumulative exposure time to *Leptospira* and the chances, therefore, are higher for older boars to have had multiple subclinical infections with different serovars. Boars that had a humoral response to *Leptospira* have the antibodies for specific serovars in their sera.

*Leptospira* detection by polymerase chain reaction (PCR) is known for its specificity and sensitivity, but it does not allow for the identification of the serovar [6,53]. To date, several studies have been performed using boar kidneys, livers, and reproduction organs [30,31,54,55,56]. It was established that in Croatia, 8.4% of boar kidneys had *Leptospira* DNA [31]; in Japan, 15.2% of boar kidneys were positive [55], while in Italy the percentage varied 11.2–12.6% for kidneys and was 30.3% for reproductive organs [30,54,56], demonstrating yet another *Leptospira* transmission method. In swine, artificial insemination is demonstrated to be an important risk factor for *Leptospira* contraction [47]. Additionally, in boars, subclinical genital leptospirosis was detected and isolated in testicles, epididymes, uteri, placentas, and fetuses [56,57]. This newly recognized spreading mechanism should be further investigated as it could explain why older, sexually mature boars tend to have a significantly higher prevalence of infection [35,36,37,42].

Panevėžys is located in northeast Lithuania, and it was the county with the highest percentage of *Leptospira* antibody-positive boars (87.9%), as well as horses [51]. Possible explanations include different climatic and socioeconomic reasons. As described by Taminskas et al., 2011 [58], Panevėžys county is located in a highly humid climate: soils there are less permeable to water and the Nevėžis river sub-basin area has the second largest expanse of wet forests (wet woodlands make up 21.5% of the territory). Abundant rivers, lakes, and wetlands create ideal conditions for leptospires to thrive in water and soil as well as for the boars to inhabit. In our study, Marijampolė county had the fewest seropositive boars (13.3%). This location has significantly fewer wetlands and wet woodlands than Panevėžys [58].

We acknowledge that our study has several limitations. MAT is a test that has a limited specificity and sensitivity. It requires careful attention and inspection to avoid false positive or false negative results [2,4,6,7,28]. However, the most important limitation of our study is that we only tested boar sera for eight serovars. It is possible, therefore, that other serovars were distributed within the boar population as well, but we failed to identify them.

## 5. Conclusions

One hundred and two (22.6%) boars had *Leptospira* antibodies in their sera. The hunted animals were mostly positive for serovars Copenhageni (9.8%), Bratislava (9.3%), and Canicola (8.2%). Boars over 24 months old had higher serological prevalence and were affected by a higher number of serovars than younger boars. The highest number of positive reactions was observed in Panevėžys (87.9%), which is known for its highly humid soil, and is rich in wet woodlands. The results of our study might indicate that boars are reservoir animals of *Leptospira* and contribute to its transmission in Lithuania.

## Figures and Tables

**Table 1:** Seropositivity to different *Leptospira* serovars in three age groups. (Pom—Pomona; Gryp—Grypothyphosa; Sax—Saxkoebing; Sej—Sejroe; Can—Canicola; Brat—Bratislava; Copen—Copenhageni; Tar—Tarassovi). Results are displayed as *n* (%, Confidence Interval of 95% (CI)).

Age of Boars	Number of Positive Reactions, *n* (%, CI)								
	Pom	Gryp	Sax *	Sej	Can *	Brat *	Copen	Tar	Total
Younger than 12 months	2 (1.0%, 0.1–3.7)	0 (0%, 0–1.9)	0 (0%, 0–1.9)	1 (0.6% 0.01–2.8)	2 (1.0%, 0.1–3.7)	2 (1.0%, 0.1–3.7)	1 (0.6%, 0.01–2.8)	2 (1.0%, 0.1–3.7)	10 (5.2%, 2.5–9.3)
12 to 24 months	4 (2.1%, 0.6–5.2)	4 (2.1%, 0.6–5.2)	2 (1.0%, 0.1–3.7)	11 (5.7%, 2.9–9.9)	12 (6.2%, 3.2–10.6)	14 (7.2%, 4.0–11.8)	22 (11.3%, 7.3–16.7)	4 (2.1%, 0.6–5.2)	73 (37.6%, 30.8–44.9)
Older than 24 months	7 (3.6%, 1.5–7.3)	8 (4.1%, 1.8–8.0)	8 (4.1%, 1.8–8.0)	9 (4.6%, 2.1–8.6)	23 (11.9%, 7.7–17.3)	26 (13.4%, 9.0–19.0)	21 (10.8%, 6.8–16.1)	9 (4.6%, 2.1–8.6)	111 (57.2%, 49.9–64.3)
Total	13 (6.7%, 3.6–11.2)	12 (6.2%, 3.2–10.6)	10 (5.2%, 2.5–9.3)	21 (10.8%, 6.8–16.1)	37 (19.1%, 13.8–25.3)	42 (21.6%, 16.1–28.1)	44 (22.7%, 17.0–29.2)	15 (7.7%, 4.4–12.4)	194 (100%)

* Difference between age groups is statistically significant (*p* < 0.05).

**Table 2:** Number of positive reactions to different *Leptospira* serovars in 10 different counties. (Pom—Pomona; Gryp—Grypothyphosa; Sax—Saxkoebing; Sej—Sejroe; Can—Canicola; Brat—Bratislava; Copen—Copenhageni; Tar—Tarassovi). Results are displayed as *n* (%, Confidence Interval of 95% (CI)).

Number of Positive Reactions, *n* (%, CI)									
County	Pom	Gryp	Sax	Sej	Can	Brat	Copen	Tar	Total
Vilnius	3 (5.5%, 1.1–15.1)	1 (1.8%, 0.1–9.7)	1 (1.8%, 0.1–9.7)	4 (7.4%, 2–17.6)	8 (14.5%, 6.5–26.7)	7 (12.7%, 5.3–24.5)	11 (20.0%, 10.4–33.0)	3 (5.5%, 1.1–15.1)	38/55 (69.1%, 55.2–80.9)
Kaunas	3 (5.0%, 1.0–13.9)	3 (5.0%, 1.0–13.9)	4 (6.7%, 1.9–16.2)	3 (5.0%, 1.0–13.9)	6 (10.0%, 3.8–20.5)	6 (10.0%, 3.8–20.5)	6 (10.0%, 3.8–20.5)	3 (5.0%, 1.0–13.9)	34/60 (56.7%, 43.2–69.4)
Klaipėda	2 (4.8%, 0.6–16.2)	0 (0%, 0–8.4)	1 (2.4%, 0.06–12.6)	3 (7.1%, 1.5–19.5)	0 (0%, 0–8.4)	3 (7.1%, 1.5–19.5)	1 (2.4%, 0.06–12.6)	1 (2.4%, 0.06–12.6)	11/42 (26.2%, 13.9–42.0)
Šiauliai	3 (5.4%, 1.1–14.9)	2 (3.6%, 0.4–12.3)	1 (1.8%, 0.04–9.6)	3 (5.4%, 1.1–14.9)	4 (7.1%, (2.0–17.3)	8 (14.3%, 6.38–26.22)	5 (8.9%, 3.0–19.6)	2 (3.6%, 0.4–12.3)	28/56 (50.0%, 36.3–63.7)
Panevėžys	2 (6.1%, 0.7–20.2)	3 (9.1%, 1.9–24.3)	1 (3.0%, 0.08–15.8)	4 (12.1%, 3.4–28.2)	5 (15.2%, 5.1–31.9)	5 (15.2%, 5.1–31.9)	4 (12.1%, 3.4–28.2)	5 (15.2%, 5.1–31.9)	29/33 (87.9%, 71.8–96.6)
Marijampolė	0 (0%, 0–21.8)	0 (0%, 0–21.8)	0 (0%, 0–21.8)	0 (0%, 0–21.8)	1 (6.7%, 0.2–32.0)	0 (0%, 0–21.8)	1 (6.7%, 0.2–32.0)	0 (0%, 0–21.8)	2/15 (13.3%, 1.7–40.5)
Telšiai	0 (0%, 0–6.4)	1 (1.8%, 0.04–9.6)	0 (0%, 0–6.4)	1 (1.8%, 0.04–9.6)	5 (8.9%, 3.0–19.6)	2 (3.6%, 0.4–12.3)	0 (0%, 0–6.4)	1 (1.8%, 0.04–9.6)	10/56 (17.9%, 8.9–30.4)
Tauragė	0 (0%, 0–8.6)	1 (2.5%, 0.06–1.9)	0 (0%, 0–8.6)	2 (4.9%, 0.6–16.5)	4 (9.8%, 2.7–23.1)	4 (9.8%, 2.7–23.1)	5 (12.2%, 4.1–26.2)	0 (0%, 0–8.6)	16/41 (39.0%, 24.2–55.5)
Alytus	0 (0%, 0–9.5)	1 (2.7%, 0.07–14.2)	1 (2.7%, 0.07–14.2)	1 (2.7%, 0.07–14.2)	2 (5.4%, 0.7–18.2)	3 (8.1%, 1.7–21.9)	1 (2.7%, 0.07–14.2)	0 (0%, 0–9.5)	9/37 (24.3%, 11.8–41.2)
Utena	0 (0%, 0–6.4)	0 (0%, 0–6.4)	1 (1.8%, 0.04–9.6)	0 (0%, 0–6.4)	2 (3.6%, 0.4–12.3)	4 (7.1%, 2.0–17.2)	10 (17.9%, 8.9–30.4)	0 (0%, 0–6.4)	17/56 (30.4%, 18.8–44.1)

**Table 3:** Seropositivity to *Leptospira* in 12 different countries, adapted from references indicated within square brackets [30,31,32,33,34,35,36,37,38,39,40,41,42,43].

Study	Country	Number of Sera	Positive Samples	% of Positive Samples	Most Common Serovar
Vale-Gonçalves, 2015 [37]	Portugal	101	66	65.4	Tarassovi
Vengust G, 2008 [41]	Slovenia	437	200	45.5	Tarassovi
Cvetnic Z, 2003 [31]	Croatia	154	40	26	Pomona
Slavica A, 2010 [35]	Croatia	351	112	31.9	Australis
Fornazari F, 2011 [33]	Brazil	308	63	20.5	Hardjo
Roquelo C, 2021 [38]	France	358	66	18.4	Australis
Jansen A, 2007 [34]	Germany	141	25	17.7	Pomona
Treml F, 2003 [36]	Czech Republic	307	52	16.9	Grippothyphosa
Espí A, 2010 [43]	Spain	171	25	14.6	Pomona
Cilia G, 2020 [30]	Italy	287	39	13.6	Australis
Chiari M, 2016 [42]	Italy	2101	321	15.3	Bratislava
Ebani VV, 2003 [32]	Italy	562	34	6	Bratislava
Żmudzki J, 2015 [39]	Poland	3621	377	10.4	Hardjo
Boqvist S, 2012 [40]	Sweden	386	12	3.1	Bratislava
Current study	Lithuania	451	102	22.6	Copenhageni

## Data Availability

Partial data is available upon request.
